# RESTORING GLUTATHIONE HAS BENEFITS FOR OLDER ADULTS AND MICE

**DOI:** 10.1093/geroni/igae098.0413

**Published:** 2024-12-31

**Authors:** George Taffet

**Affiliations:** Chair

## Abstract

The role and importance of mitochondrial function and glutathione is not well recognized by clinicians Glutathione is the most abundant intracellular anti-oxidant and is responsible for detoxifying many reactive, potentially harmful molecules and is most older adults are glutathione deficient because of low free Cysteine and especially Glycine. Supplementation with Glycine and N-Acetyl Cysteine is able to restore cellular glutathione. This symposium will fill these gaps by providing knowledge about glutathione, its role in defending anti-oxidant status, the deficient precursors, and the results when glutathione is restored by providing the precursors and the potential sex differences in these effects.

